# The chromosomal genome sequence of the marine sponge *Diacarnus erythraeanus *Kelly-Borges & Vacelet, 1995, and its associated microbial metagenome sequences

**DOI:** 10.12688/wellcomeopenres.24763.1

**Published:** 2025-08-28

**Authors:** Laura Steindler, Martha Alejandra Durán Canché, Micha Ilan, Rinat Bar-Shalom, Jose Victor Lopez, Ute Hentschel, Graeme Oatley, Elizabeth Sinclair, Eerik Aunin, Noah Gettle, Camilla Santos, Michael Paulini, Haoyu Niu, Victoria McKenna, Rebecca O’Brien, Wellcome Sanger Institute Tree of Life Management, Samples and Laboratory Team, Wellcome Sanger Institute Scientific Operations: Sequencing Operations, Wellcome Sanger Institute Tree of Life Core Informatics Team, EBI Aquatic Symbiosis Genomics Data Portal Team, Aquatic Symbiosis Genomics Project Leadership

**Affiliations:** 1 University of Haifa, Haifa, Haifa District, Israel; 2 Tel Aviv University, Tel Aviv, Tel Aviv District, Israel; 3 National Coral Reef Institute, Department of Biological Sciences, Nova Southeastern University, Dania Beach, Florida, USA; 4 GEOMAR Helmholtz Centre for Ocean Research Kiel, Kiel, Germany; 5 Tree of Life, Wellcome Sanger Institute, Hinxton, England, UK

**Keywords:** Diacarnus erythraeanus, sponge, genome sequence, chromosomal, Poecilosclerida (older classification), Bubarida (new classification), microbial metagenome

## Abstract

We present a genome assembly from an individual *Diacarnus erythraeanus* (sponge; Porifera; Demospongiae; Poecilosclerida; Podospongiidae). The genome sequence has a total length of 140.86 megabases. Most of the assembly (98.57%) is scaffolded into 18 chromosomal pseudomolecules. The mitochondrial genome has also been assembled and is 19.34 kilobases in length. Sixty-four binned genomes were generated from the metagenome assembly, of which 46 were classified as high-quality metagenome assembled genomes (MAGs). The microbial signature is typical of HMA sponges, including the Pseudomonadota, Chloroflexota and Acidobacteriota as dominant phyla and several candidate phyla (Poribacteria, Binatota, Latescibacterota) as well as the archaeal clade Nitrosopumilaceae in lower abundance.

## Species taxonomy

Eukaryota; Opisthokonta; Metazoa; Porifera; Demospongiae; Heteroscleromorpha; Poecilosclerida; Podospongiidae; *Diacarnus*; *Diacarnus erythraeanus* Kelly-Borges & Vacelet, 1995 (NCBI:txid879318)

## Background

*Diacarnus erythraeanus* Kelly-Borges & Vacelet, 1995, is a marine sponge endemic to the Red Sea. It is usually found on sandy substrates or attached to rocks and small corals in well-lit areas ( Moskovich *et al.*, 2023). This species thrives at depths ranging from 10 to 30 m ( Kelly-Borges & Vacelet, 1995), although in Eilat it has also been observed in shallower and deeper waters (2–10 and 40–55 m, respectively; Raijman-Nagar *et al.*, 2023). The sponge is characterised by its repent or erect thick branches, which range from 20 to 50 mm in diameter. The branching pattern varies among specimens, and its colouration transitions from rose pink, with mottled oak brown on the surface, to a darker shade near concave depressions and with tips of the branches turning uniformly brown. The species is easily recognisable in the field due to its rubbery texture. The primary fibres, up to 1.8 mm in diameter, are radially arranged toward the sponge’s surface and are typically adjacent to secondary fibres measuring 270–420 µm in diameter.

The individuals are viviparous hermaphrodites, exhibiting no defined reproductive season throughout the year ( Oren *et al.*, 2005). This species shows asynchronous reproduction, distinguished by yellow-coloured embryos and incubated larvae that are large enough to be visible to the naked eye, usually embedded in collagenic niches within the sponge mesohyl. During early development, the larvae form a spherical shape covered with cilia. As they mature, they elongate, developing into free-swimming, fully ciliated parenchymula larvae, dark yellow in colour and approximately 5 mm in length. It is interesting to note that barnacles ( *Acasta pertusa*) residing in *D. erythraenus* specimens were found to contain embryos all year round ( Ilan *et al.*, 1999). The presence of cyanobacteria has been documented not only in adult specimens of *D. erythraenus* but also within its larvae ( Oren *et al.*, 2005), indicating the vertical transmission of symbionts in this species ( Carrier *et al.*, 2022). Beyond cyanobacteria, additional bacterial lineages have been identified in both adult and larval specimens of *D. erythraenus.* These include members of the Pseudomonadota, the candidate phylum Poribacteria, Chloroflexota, Nitrospirota and Bacteroidetes ( Bergman *et al.*, 2011). In both shallow and mesophotic populations, the bacterial community within *D. erythraenus* is highly dynamic and responsive to environmental stress. Long-term exposure to acute high temperatures was shown to lead to shifts in bacterial abundance, reflecting stress responses and adaptation to heat ( Raijman-Nagar *et al.*, 2023).

One remarkable trait of *Diacarnus* is its ability to produce secondary metabolites, many of which show potential for medical applications. For example, recent research has revealed that this genus produces a variety of peroxide compounds derived from terpenes, which have demonstrated effectiveness against tumour cells ( Lefranc *et al.*, 2013; Torres-García *et al.*, 2021). Additionally, norterpene derivatives isolated from *D. megaspinorhabdosa* have exhibited substantial activities against carcinomas, lymphoma, and brain cell tumours ( Ibrahim *et al.*, 2008). Other terpene-related metabolites have also been identified; for example, muqubilone has shown antimalarial activity against *Plasmodium falciparum*, while both muquibilin and muqubilone have demonstrated antiviral properties when tested against the herpes virus ( El Sayed *et al.*, 2001).

From an ecological perspective, secondary metabolites offer only partial protection for *D. erythraenus* against its predators, providing moderate deterrence against sea urchins such as *Diadema setosum*. However, the species is primarily preyed upon by fish, *Thalassoma klunzingeri* ( Burns *et al.*, 2003). The impact of these metabolites on the sponge’s palatability can vary, even between specific predator-prey interactions. While *T. klunzingeri* appears to tolerate the sponge’s defences, *Thalassoma pavo,* is deterred by the same chemical compounds ( Sokolover & Ilan, 2007).

Similar to other sponge species, *D. erythraeanus* accumulates trace metals. It has been suggested that its spicules may act as sinks for boron, while cadmium is directly stored in the sponge’s tissue. Copper accumulation is a highly species-specific process, likely occurring through the uptake of seawater, whereas zinc is actively accumulated, possibly through the consumption of particulate organic matter (POM) ( Mayzel *et al.*, 2014).

**Figure 1.**
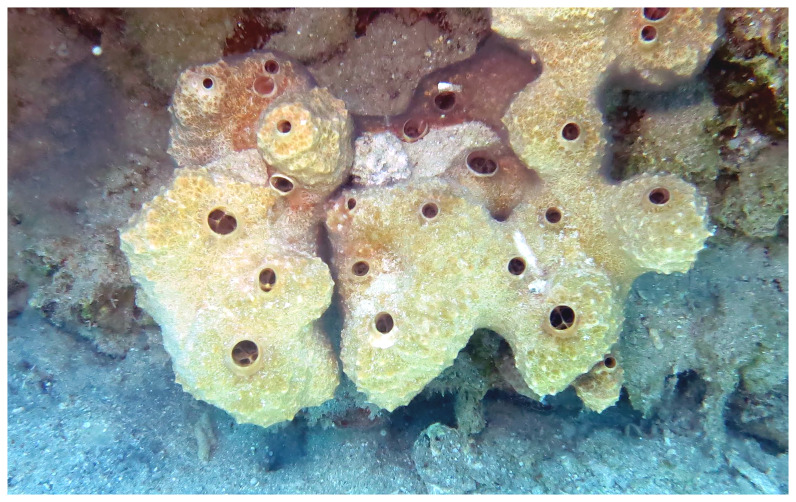
Photograph of the *Diacarnus erythraeanus* (odDiaEryt1) specimen used for genome sequencing.

Information on *D. erythraeanus* is limited, with research primarily centred on the isolation and identification of secondary metabolites with biotechnological potential. Access to genomic data on both sponge and its associated symbionts will enhance our understanding of the metabolic pathways underlying the holobiont’s ability to produce bioactive compounds. We present a chromosomally complete genome sequence for *Diacarnus erythraeanus*, based on a specimen from Eilat, Israel ( Figure 1).

## Genome sequence report

### Sequencing data

The genome of a specimen of *Diacarnus erythraeanus* ( Figure 1) was sequenced using Pacific Biosciences single-molecule HiFi long reads, generating 77.20 Gb from 10.22 million reads. Based on the estimated genome size, the sequencing data provided approximately 296× coverage of the genome. Chromosome conformation Hi-C data produced 136.11 Gb from 901.40 million reads. RNA sequencing data were also generated and are available in public sequence repositories. Table 1 summarises the specimen and sequencing information.

**Table 1.** Specimen and sequencing data for *Diacarnus erythraeanus*.

Project information			
**Study title**	Diacarnus erythraeanus		
**Umbrella BioProject**	PRJEB72481		
**Species**	*Diacarnus erythraeanus*		
**BioSpecimen**	SAMEA8712135		
**NCBI taxonomy ID**	879318		
Specimen information			
**Technology**	**ToLID**	**BioSample ** **accession**	**Organism part**
**PacBio long read sequencing**	odDiaEryt1	SAMEA8712223	Somatic tissue
**Hi-C sequencing**	odDiaEryt1	SAMEA8712223	Somatic tissue
**RNA sequencing**	odDiaEryt1	SAMEA8712225	Somatic tissue
**Sequencing information**			
**Platform**	**Run accession**	**Read count**	**Base count (Gb)**
**Hi-C Illumina NovaSeq X**	ERR12743772	9.01e+08	136.11
**PacBio Revio**	ERR12666652	1.02e+07	77.2
**RNA Illumina NovaSeq X**	ERR13669964	1.09e+08	16.43

### Assembly statistics

The primary haplotype was assembled, and contigs corresponding to an alternate haplotype were also deposited in INSDC databases. The assembly was improved by manual curation, which corrected 85 misjoins or missing joins and removed 11 haplotypic duplications. These interventions reduced the total assembly length by 24.61%, decreased the scaffold count by 68.6%, and increased the scaffold N50 by 8.45%. The final assembly has a total length of 140.86 Mb in 145 scaffolds, with 199 gaps, and a scaffold N50 of 7.46 Mb ( Table 2).

**Table 2.** Genome assembly data for *Diacarnus erythraeanus*.

Genome assembly	
Assembly name	odDiaEryt1.1
Assembly accession	GCA_964016965.1
*Alternate haplotype accession*	*GCA_964016915.1*
Assembly level for primary assembly	chromosome
Span (Mb)	140.86
Number of contigs	344
Number of scaffolds	145
Longest scaffold (Mb)	10.91
Assembly metric	Measure
Contig N50 length	1.39 Mb
Scaffold N50 length	7.46 Mb
Consensus quality (QV)	Primary: 55.2; alternate: 51.5; combined 52.2
BUSCO *	C:79.0%[S:78.3%,D:0.7%],F:8.6%,M:12.4%,n:954
Percentage of assembly assigned to chromosomes	98.33%
Organelles	Mitochondrial genome: 19.34 kb

* BUSCO scores based on the metazoa_odb10 BUSCO set using version 5.5.0. C = complete [S = single copy, D = duplicated], F = fragmented, M = missing, n = number of orthologues in comparison.

**Figure 2.**
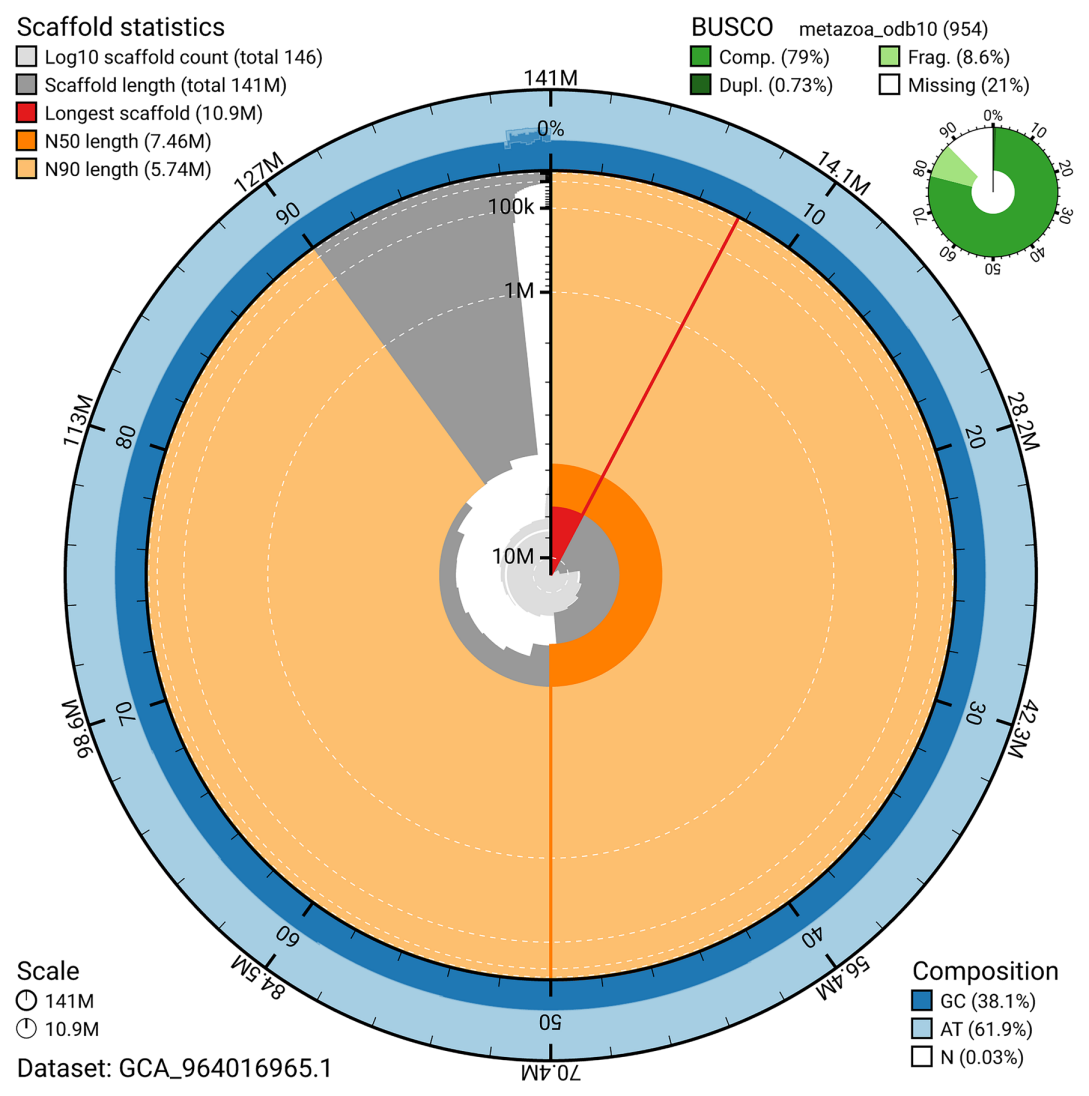
Genome assembly of *Diacarnus erythraeanus*, odDiaEryt1.1: metrics. The BlobToolKit snail plot provides an overview of assembly metrics and BUSCO gene completeness. The circumference represents the length of the whole genome sequence, and the main plot is divided into 1,000 bins around the circumference. The outermost blue tracks display the distribution of GC, AT, and N percentages across the bins. Scaffolds are arranged clockwise from longest to shortest and are depicted in dark grey. The longest scaffold is indicated by the red arc, and the deeper orange and pale orange arcs represent the N50 and N90 lengths. A light grey spiral at the centre shows the cumulative scaffold count on a logarithmic scale. A summary of complete, fragmented, duplicated, and missing BUSCO genes in the metazoa_odb10 set is presented at the top right. An interactive version of this figure is available at https://blobtoolkit.genomehubs.org/view/GCA_964016965.1/dataset/GCA_964016965.1/snail.

**Figure 3.**
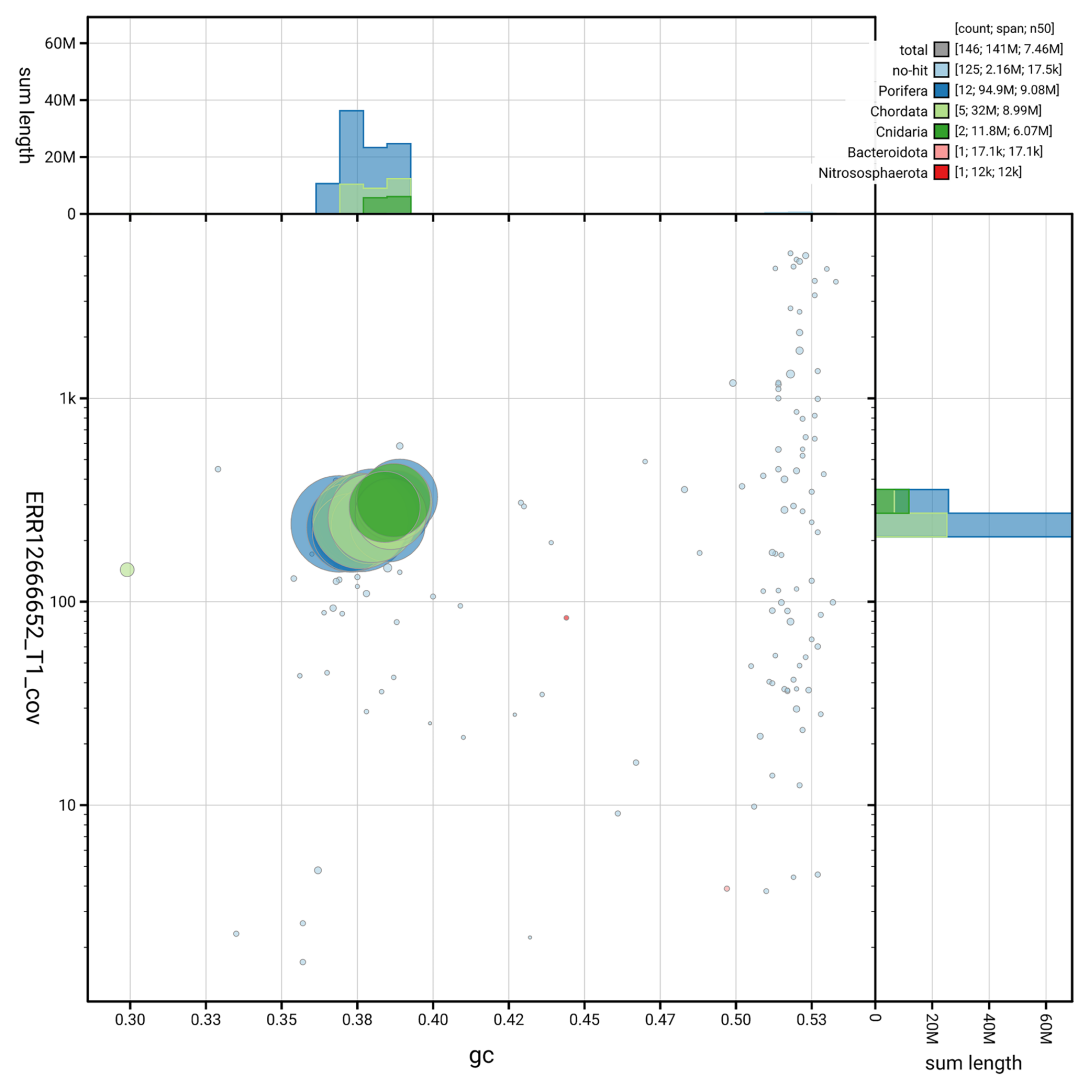
Genome assembly of *Diacarnus erythraeanus*, odDiaEryt1.1: BlobToolKit GC-coverage plot. Blob plot showing sequence coverage (vertical axis) and GC content (horizontal axis). The circles represent scaffolds, with the size proportional to scaffold length and the colour representing phylum membership. The histograms along the axes display the total length of sequences distributed across different levels of coverage and GC content. An interactive version of this figure is available at https://blobtoolkit.genomehubs.org/view/GCA_964016965.1/dataset/GCA_964016965.1/blob.

**Figure 4.**
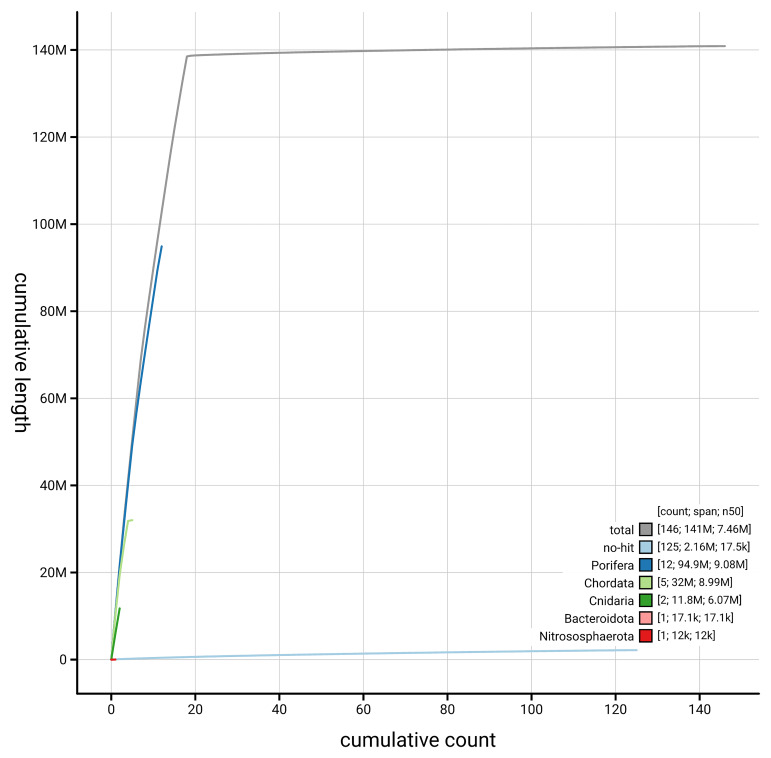
Genome assembly of *Diacarnus erythraeanus,* odDiaEryt1.1: BlobToolKit cumulative sequence plot. The grey line shows cumulative length for all scaffolds. Coloured lines show cumulative lengths of scaffolds assigned to each phylum using the buscogenes taxrule. An interactive version of this figure is available at https://blobtoolkit.genomehubs.org/view/GCA_964016965.1/dataset/GCA_964016965.1/cumulative.

The snail plot in Figure 2 provides a summary of the assembly statistics, indicating the distribution of scaffold lengths and other assembly metrics. Figure 3 shows the distribution of scaffolds by GC proportion and coverage. Figure 4 presents a cumulative assembly plot, with separate curves representing different scaffold subsets assigned to various phyla, illustrating the completeness of the assembly.

**Figure 5.**
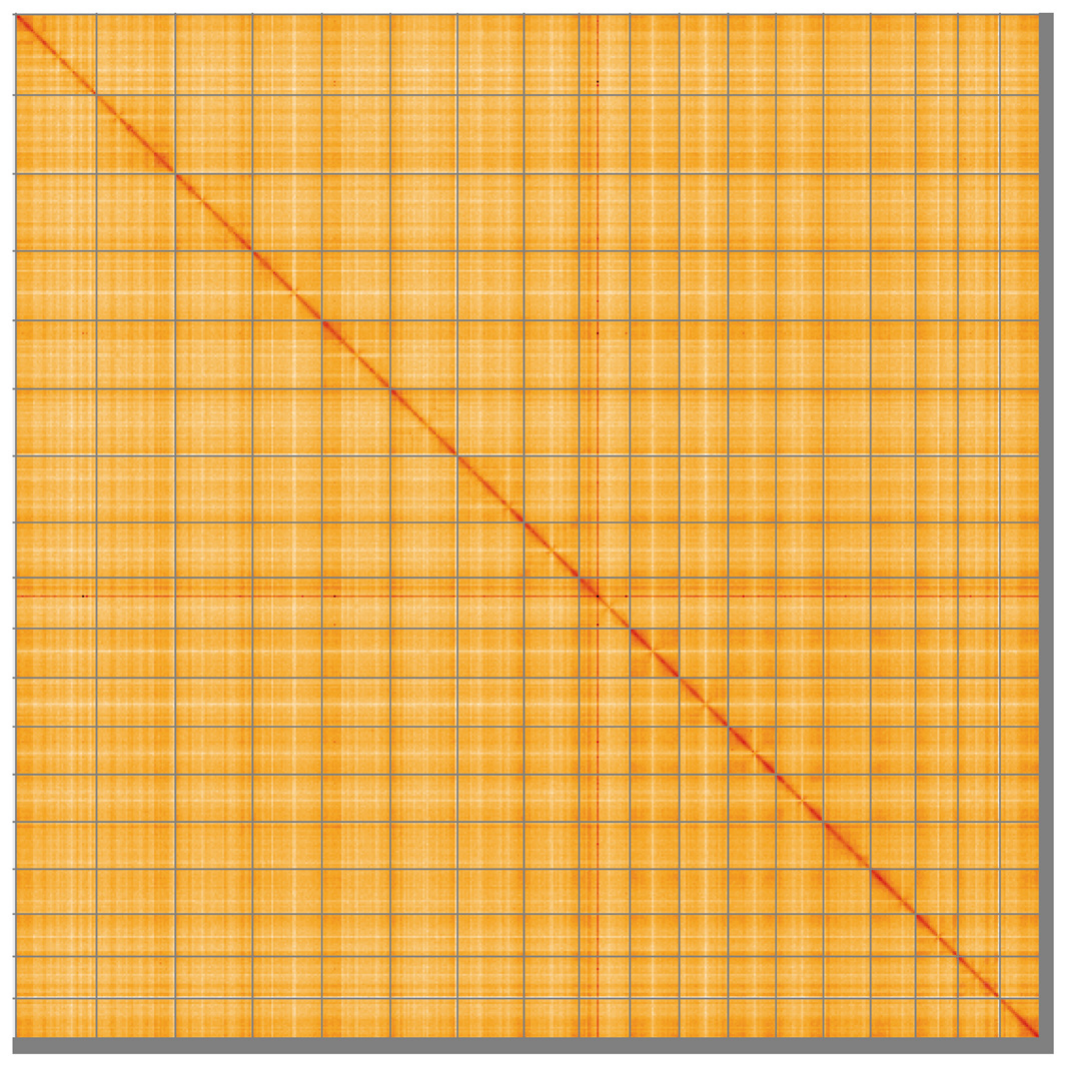
Genome assembly of *Diacarnus erythraeanus*: Hi-C contact map of the odDiaEryt1.1 assembly, visualised using HiGlass. Chromosomes are shown in order of size from left to right and top to bottom. An interactive version of this figure may be viewed at https://genome-note-higlass.tol.sanger.ac.uk/l/?d=Tnm0QBYEQF2fSRwWbpf3GA.

Most of the assembly sequence (98.33%) was assigned to 18 chromosomal-level scaffolds. These chromosome-level scaffolds, confirmed by Hi-C data, are named according to size ( Figure 5; Table 3).

**Table 3.** Chromosomal pseudomolecules in the genome assembly of *Diacarnus erythraeanus*, odDiaEryt1.

INSDC accession	Name	Length (Mb)	GC%
OZ024669.1	1	10.91	37.5
OZ024670.1	2	10.66	37
OZ024671.1	3	10.44	37.5
OZ024672.1	4	9.39	37.5
OZ024673.1	5	9.27	37.5
OZ024674.1	6	9.08	37.5
OZ024675.1	7	8.99	38
OZ024676.1	8	7.46	38
OZ024677.1	9	6.88	37.5
OZ024678.1	10	6.67	38.5
OZ024679.1	11	6.47	39
OZ024680.1	12	6.61	38
OZ024681.1	13	6.41	38.5
OZ024682.1	14	6.39	38.5
OZ024683.1	15	6.07	38.5
OZ024684.1	16	5.74	38.5
OZ024685.1	17	5.69	38.5
OZ024686.1	18	5.37	38.5
OZ024687.1	MT	0.02	33

The mitochondrial genome was also assembled. This sequence is included as a contig in the multifasta file of the genome submission and as a standalone record in GenBank.

### Assembly quality metrics

The combined primary and alternate assemblies achieve an estimated Quality Value (QV) of 52.2. BUSCO v.5.5.0 analysis using the metazoa_odb10 reference set ( *n* = 954) identified 79.0% of the expected gene set (single = 78.3%, duplicated = 0.7%).

## Metagenome report

**Figure 6.**
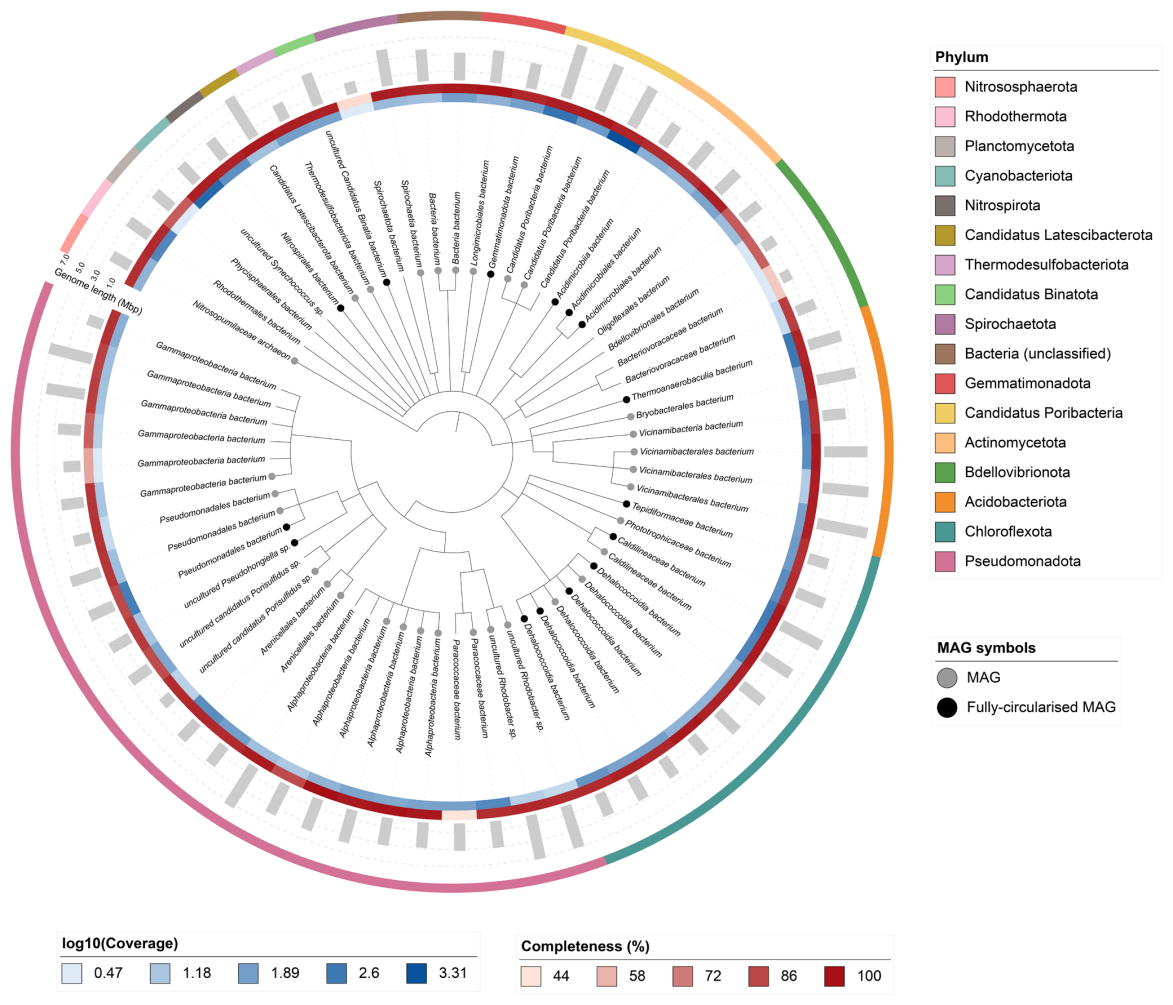
Cladogram showing the taxonomic placement of metagenome bins, constructed using NCBI taxonomic identifiers with *taxonomizr* and annotated in iTOL. Colours indicate phylum-level taxonomy. Additional tracks show sequencing coverage (log _10_), genome size (Mbp), and completeness (%). Bins that meet the criteria for MAGs are marked with a grey circle; fully circularised MAGs are marked in black.

Sixty-four binned genomes were generated from the metagenome assembly ( Figure 6), of which 46 were classified as high-quality metagenome assembled genomes (MAGs) (see methods). The completeness values for these assemblies range from approximately 45% to 100% with contamination below 9%. A cladogram of the binned metagenomes is shown in Figure 6. For details on binned genomes see https://doi.org/10.6084/m9.figshare.29695256.v1.

## Methods

### Sample acquisition

A *Diacarnus erythraeanus* specimen (specimen ID NSU0043101, ToLID odDiaEryt1) was collected on 2013-12-23 from Eilat, Israel (latitude 29.501, longitude 34.917) from a depth of 12 m by SCUBA diving. The specimen was collected by Maya Britstein and identified by Rinat Bar-Shalom and Ilia Burgsdorf (University of Haifa, Israel) and preserved by snap-freezing.

### Nucleic acid extraction

The workflow for high molecular weight (HMW) DNA extraction at the Wellcome Sanger Institute (WSI) Tree of Life Core Laboratory includes a sequence of procedures: sample preparation and homogenisation, DNA extraction, fragmentation and purification. Detailed protocols are available on protocols.io ( Denton *et al.*, 2023). The odDiaEryt1 sample was prepared for DNA extraction by weighing and dissecting it on dry ice ( Jay *et al.*, 2023). Prior to DNA extraction, the sponge sample was bathed in “L buffer” (10 mM Tris, pH 7.6, 100 mM EDTA, 20 mM NaCl), minced into small pieces using a scalpel and the cellular interior separated from the mesohyl using forceps ( Lopez, 2022). HMW DNA was extracted using the Automated MagAttract v2 protocol ( Oatley *et al.*, 2023a). DNA was sheared into an average fragment size of 12–20 kb in a Megaruptor 3 system ( Bates *et al.*, 2023). Sheared DNA was purified by solid-phase reversible immobilisation, using AMPure PB beads to eliminate shorter fragments and concentrate the DNA ( Oatley *et al.*, 2023b). The concentration of the sheared and purified DNA was assessed using a Nanodrop spectrophotometer and Qubit Fluorometer using the Qubit dsDNA High Sensitivity Assay kit. Fragment size distribution was evaluated by running the sample on the FemtoPulse system.

RNA was also extracted from tissue of odDiaEryt1 in the Tree of Life Laboratory at the WSI using the RNA Extraction: Automated MagMax™ *mir*Vana protocol ( do Amaral *et al.*, 2023). The RNA concentration was assessed using a Nanodrop spectrophotometer and a Qubit Fluorometer using the Qubit RNA Broad-Range Assay kit. Analysis of the integrity of the RNA was done using the Agilent RNA 6000 Pico Kit and Eukaryotic Total RNA assay.

### Sequencing

Pacific Biosciences HiFi circular consensus DNA sequencing libraries were constructed according to the manufacturers’ instructions. DNA sequencing was performed by the Scientific Operations core at the WSI on the Pacific Biosciences Revio instrument. Somatic tissue from the odDiaEryt1 sample was processed for Hi-C sequencing at the WSI Scientific Operations core, using the Arima-HiC v2 kit and sequenced on the Illumina NovaSeq X instrument. Poly(A) RNA-Seq libraries were constructed using the NEB Ultra II RNA Library Prep kit, following the manufacturer’s instructions. RNA sequencing was performed on the Illumina NovaSeq X instrument.

### Genome assembly, curation and evaluation

***Assembly***

Prior to assembly of the PacBio HiFi reads, a database of *k*-mer counts ( *k* = 31) was generated from the filtered reads using FastK. GenomeScope2 ( Ranallo-Benavidez *et al.*, 2020) was used to analyse the *k*-mer frequency distributions, providing estimates of genome size, heterozygosity, and repeat content.

The HiFi reads were assembled using Hifiasm ( Cheng *et al.*, 2021) with the --primary option. Haplotypic duplications were identified and removed using purge_dups ( Guan *et al.*, 2020). The Hi-C reads were mapped to the primary contigs using bwa-mem2 ( Vasimuddin *et al.*, 2019). The contigs were further scaffolded using the provided Hi-C data ( Rao *et al.*, 2014) in YaHS ( Zhou *et al.*, 2023) using the --break option for handling potential misassemblies. The scaffolded assemblies were evaluated using Gfastats ( Formenti *et al.*, 2022), BUSCO ( Manni *et al.*, 2021) and MERQURY.FK ( Rhie *et al.*, 2020).

The mitochondrial genome was assembled using MitoHiFi ( Uliano-Silva *et al.*, 2023), which runs MitoFinder ( Allio *et al.*, 2020) and uses these annotations to select the final mitochondrial contig and to ensure the general quality of the sequence.

***Assembly curation***

The assembly was decontaminated using the Assembly Screen for Cobionts and Contaminants (ASCC) pipeline. Flat files and maps used in curation were generated via the TreeVal pipeline ( Pointon *et al.*, 2023). Manual curation was conducted primarily in PretextView ( Harry, 2022) and HiGlass ( Kerpedjiev *et al.*, 2018), with additional insights provided by JBrowse2 ( Diesh *et al.*, 2023). Scaffolds were visually inspected and corrected as described by Howe *et al*. (2021). Any identified contamination, missed joins, and mis-joins were amended, and duplicate sequences were tagged and removed. The curation process is documented at https://gitlab.com/wtsi-grit/rapid-curation.

***Assembly quality assessment***

The Merqury.FK tool ( Rhie *et al.*, 2020), run in a Singularity container ( Kurtzer *et al.*, 2017), was used to assembly quality for the primary and alternate haplotypes using the *k*-mer databases ( *k* = 31) that were computed prior to genome assembly.

A Hi-C contact map was produced for the final version of the assembly. The Hi-C reads were aligned using bwa-mem2 ( Vasimuddin *et al.*, 2019) and the alignment files were combined using SAMtools ( Danecek *et al.*, 2021). The Hi-C alignments were converted into a contact map using BEDTools ( Quinlan & Hall, 2010) and the Cooler tool suite ( Abdennur & Mirny, 2020). The contact map is visualised in HiGlass ( Kerpedjiev *et al.*, 2018).

The genome was analysed using the BlobToolkit pipeline, a Nextflow port of the earlier Snakemake version ( Challis *et al.*, 2020). The pipeline aligns PacBio reads using SAMtools and minimap2 ( Li, 2018) and generates coverage tracks for regions of fixed size. It runs BUSCO ( Manni *et al.*, 2021) using lineages identified from the NCBI taxonomy ( Schoch *et al.*, 2020). For the three domain-level lineages, BUSCO genes are aligned to the UniProt Reference Proteomes ( Bateman *et al.*, 2023) using DIAMOND blastp ( Buchfink *et al.*, 2021). The genome is divided into chunks according to the density of the BUSCO genes from the closest taxonomic lineage, and each chunk is aligned to the UniProt Reference Proteomes using DIAMOND blastx. Genome sequences without a hit are split using seqtk and aligned to the NT database with blastn ( Altschul *et al.*, 1990). The blobtools suite consolidates all outputs into a blobdir for visualisation. The BlobToolKit pipeline was developed using nf-core tooling ( Ewels *et al.*, 2020) and MultiQC ( Ewels *et al.*, 2016), with containerisation through Docker ( Merkel, 2014) and Singularity ( Kurtzer *et al.*, 2017).

Table 4 contains a list of relevant software tool versions and sources.

**Table 4.** Software tools: versions and sources.

Software tool	Version	Source
BEDTools	2.30.0	https://github.com/arq5x/bedtools2
bin3C	0.3.3	https://github.com/cerebis/bin3C
BLAST	2.14.0	ftp://ftp.ncbi.nlm.nih.gov/blast/executables/blast+/
BlobToolKit	4.3.9	https://github.com/blobtoolkit/blobtoolkit
BUSCO	5.5.0	https://gitlab.com/ezlab/busco
bwa-mem2	2.2.1	https://github.com/bwa-mem2/bwa-mem2
CheckM	1.2.1	https://github.com/Ecogenomics/CheckM
Cooler	0.8.11	https://github.com/open2c/cooler
DAS Tool	-	https://github.com/cmks/DAS_Tool
DIAMOND	2.1.8	https://github.com/bbuchfink/diamond
dRep	3.4.0	https://github.com/MrOlm/drep
fasta_windows	0.2.4	https://github.com/tolkit/fasta_windows
FastK	1.1	https://github.com/thegenemyers/FASTK
gEVAL	N/A	https://geval.org.uk/
Gfastats	1.3.6	https://github.com/vgl-hub/gfastats
GoaT CLI	0.2.5	https://github.com/genomehubs/goat-cli
GTDB-TK	2.3.2	https://github.com/Ecogenomics/GTDBTk
Hifiasm	0.19.8-r603	https://github.com/chhylp123/hifiasm
HiGlass	1.13.4	https://github.com/higlass/higlass
MAGScoT	1.0.0	https://github.com/ikmb/MAGScoT
MaxBin	2.7	https://sourceforge.net/projects/maxbin/
MerquryFK	1.1	https://github.com/thegenemyers/MERQURY.FK
MetaBat2	2.15-15-gd6ea400	https://bitbucket.org/berkeleylab/metabat/src/master/
metaMDBG	Pre-release	https://github.com/GaetanBenoitDev/metaMDBG
MetaTOR	-	https://github.com/koszullab/metaTOR
Minimap2	2.24-r1122	https://github.com/lh3/minimap2
MitoHiFi	2	https://github.com/marcelauliano/MitoHiFi
MultiQC	1.14, 1.17, and 1.18	https://github.com/MultiQC/MultiQC
Nextflow	23.04.1	https://github.com/nextflow-io/nextflow
PretextView	0.2.5	https://github.com/sanger-tol/PretextView
PROKKA	1.14.5	https://github.com/vdejager/prokka
purge_dups	1.2.3	https://github.com/dfguan/purge_dups
samtools	1.19.2	https://github.com/samtools/samtools
sanger-tol/ascc	-	https://github.com/sanger-tol/ascc
sanger-tol/ blobtoolkit	0.4.0	https://github.com/sanger-tol/blobtoolkit
Seqtk	1.3	https://github.com/lh3/seqtk
Singularity	3.9.0	https://github.com/sylabs/singularity
TreeVal	1.2.0	https://github.com/sanger-tol/treeval
YaHS	1.1a.2	https://github.com/c-zhou/yahs
bin3C	0.3.3	https://github.com/cerebis/bin3C
CheckM	1.2.1	https://github.com/Ecogenomics/CheckM
dRep	3.4.0	https://github.com/MrOlm/drep
GTDB-TK	2.3.2	https://github.com/Ecogenomics/GTDBTk
MAGScoT	1.0.0	https://github.com/ikmb/MAGScoT
MaxBin	2.7	https://sourceforge.net/projects/maxbin/
MetaBat2	2.15-15-gd6ea400	https://bitbucket.org/berkeleylab/metabat/src/master/
metaMDBG	-	https://github.com/GaetanBenoitDev/metaMDBG
PROKKA	1.14.5	https://github.com/vdejager/prokka

### Metagenome assembly

The metagenome assembly was generated using MetaMDBG ( Benoit *et al.*, 2024) and binned using MetaBAT2 ( Kang *et al.*, 2019), MaxBin ( Wu *et al.*, 2014), bin3C ( DeMaere & Darling, 2019), and MetaTOR. The resulting bin sets of each binning algorithm were optimised and refined using MAGScoT ( Rühlemann *et al.*, 2022). PROKKA ( Seemann, 2014) was used to identify tRNAs and rRNAs in each bin, CheckM ( Parks *et al.*, 2015) (checkM_DB release 2015-01-16) was used to assess bin completeness/contamination, and GTDB-TK ( Chaumeil *et al.*, 2022) (GTDB release 214) was used to taxonomically classify bins. Taxonomic replicate bins were identified using dRep ( Olm *et al.*, 2017) with default settings (95% ANI threshold). The final bin set was filtered for bacteria and archaea. All bins were assessed for quality and categorised as metagenome-assembled genomes (MAGs) if they met the following criteria: contamination ≤ 5%, presence of 5S, 16S, and 23S rRNA genes, at least 18 unique tRNAs, and either ≥ 90% completeness or ≥ 50% completeness with fully circularised chromosomes. Bins that did not meet these thresholds, or were identified as taxonomic replicates of MAGs, were retained as ‘binned metagenomes’ provided they had ≥ 50% completeness and ≤ 10% contamination. A cladogram based on NCBI taxonomic assignments was generated using the ‘taxonomizr’ package in R. The tree was visualised and annotated using iTOL ( Letunic & Bork, 2024). Software tool versions and sources are given in Table 4.

### Wellcome Sanger Institute – Legal and Governance

The materials that have contributed to this genome note have been supplied by a Tree of Life collaborator. The Wellcome Sanger Institute employs a process whereby due diligence is carried out proportionate to the nature of the materials themselves, and the circumstances under which they have been/are to be collected and provided for use. The purpose of this is to address and mitigate any potential legal and/or ethical implications of receipt and use of the materials as part of the research project, and to ensure that in doing so we align with best practice wherever possible. The overarching areas of consideration are:

•     Ethical review of provenance and sourcing of the material

•     Legality of collection, transfer and use (national and international)

Each transfer of samples is undertaken according to a Research Collaboration Agreement or Material Transfer Agreement entered into by the Tree of Life collaborator, Genome Research Limited (operating as the Wellcome Sanger Institute) and in some circumstances other Tree of Life collaborators.

## Data Availability

European Nucleotide Archive: Diacarnus erythraeanus. Accession number PRJEB72481; https://identifiers.org/ena.embl/PRJEB72481. The genome sequence is released openly for reuse. The *Diacarnus erythraeanus* genome sequencing initiative is part of the Aquatic Symbiosis Genomics (ASG) project ( https://www.ebi.ac.uk/ena/browser/view/PRJEB43743). All raw sequence data and the assembly have been deposited in INSDC databases. The genome will be annotated using available RNA-Seq data and presented through the Ensembl pipeline at the European Bioinformatics Institute. Raw data and assembly accession identifiers are reported in Table 1 and Table 2.

## Author information

Contributors are listed at the following links:

- Wellcome Sanger Institute Tree of Life Management, Samples and Laboratory team
- Wellcome Sanger Institute Scientific Operations – Sequencing Operations
- Wellcome Sanger Institute Tree of Life Core Informatics team
- Tree of Life Core Informatics collective
- European Bioinformatics Institute ASG Data Portal team
- Wellcome Sanger Institute/Aquatic Symbiosis Genomics Project Leadership
