# Supplementary material for: The chromosomal genome sequence of the marine sponge Diacarnus erythraeanus Kelly-Borges & Vacelet, 1995, and its associated microbial metagenome sequences
Source: Wellcome Open Res. 2026 Apr 13;10:466. Originally published 2025 Aug 28. [Version 2] doi: 10.12688/wellcomeopenres.24763.2 (PMC13087572; doi:10.12688/wellcomeopenres.24763.2)
Supplement: Peer review history

# Reviewer report: Reviewer response for version 2

**DOI:** 10.21956/wellcomeopenres.29041.r153274

**Published:** 2026-04-16

**Authors:** Emily C Giles

**Affiliations:** 1 Cawthron Institute, Nelson, Nelson, New Zealand

The authors present nuclear, mitochondrial, and associated bacterial genome assemblies for the marine sponge Diacarnus erythraeanus. They provide a nice introduction to provide context for their study. The highlight the interesting reproductive biology and chemistry of the species. The authors generated the assemblies using PacBio HiFi (250+ coverage) and HiC for scaffolding. The same individual specimen was used for all sequencing, and the nuclear assembly (98%) resided in 18 pseudo chromosomes. The authors present 46MAGs. My only reservations about these materials include A.) the low BUSCO scores of the nuclear assembly, B.) representation of MAGs as high quality, and C.) description of the curation methods. Below I address each of these points:

 A.) The complete BUSCOs are 79% and fragmented BUSCOs are 8.6%. This could be a reflection of the deep evolutionary history of sponges, but this could also be due to issues with the assembly/ curation. How do the BUSCOs of the primary assembly compare to the BUSCOs of the final assembly? The authors could provide context for how the low complete BUSCOs compare to complete BUSCOs of other sponges. The authors could explore whether the 21% unrecovered complete BUSCOs and/or fragmented BUSCOs can be manually rescued from the assembly. Were complete BUSCOs lost during curation?

 B.) It is very difficult to glean MAG completeness from Fig 6, this information could be presented in a table. MAGs with low completeness should not be referred to as high quality.

 C.) A formal description of the rapid curation methods would be a valuable resource for the scientific community. In line with point A.) it is unclear exactly what criteria were used for curation and how this could have affected genome completeness.

 Despite these reservations the assemblies are sound and present valuable resources for sponge genomics.

Are sufficient details of methods and materials provided to allow replication by others?

Partly

Is the rationale for creating the dataset(s) clearly described?

Yes

Are the datasets clearly presented in a useable and accessible format?

Yes

Are the protocols appropriate and is the work technically sound?

Yes

Reviewer Expertise:

population genomics, comparative genomics, marine biology

I confirm that I have read this submission and believe that I have an appropriate level of expertise to confirm that it is of an acceptable scientific standard.

# Reviewer report: Reviewer response for version 1

**DOI:** 10.21956/wellcomeopenres.27280.r131107

**Published:** 2025-09-17

**Authors:** Estelle Proux-Wéra

**Affiliations:** 1 Department of Biochemistry and Biophysics, Stockholm University, stockholm, Finland

The authors present the nuclear genome and mitochondrial genome of the marine sponge *Diacarnus erythraeanus*. Sponge genomes can be difficult to assemble due to their high number of bacterial symbionts.

 The authors have used state of the art methodologies for genome assembly, curation and quality assessment, and for the metagenome assembly. The metrics are in line with other available sponge data. The methods are detailed and the data is useable and accessible. 

 One extra comment, maybe out of scope, about mitochondrial gene order: does it correspond to what is expected for Poecilosclerida (Plese et al. 2021)?

Are sufficient details of methods and materials provided to allow replication by others?

Yes

Is the rationale for creating the dataset(s) clearly described?

Yes

Are the datasets clearly presented in a useable and accessible format?

Yes

Are the protocols appropriate and is the work technically sound?

Yes

Reviewer Expertise:

Genome assembly, comparative genomics

I confirm that I have read this submission and believe that I have an appropriate level of expertise to confirm that it is of an acceptable scientific standard.

## References

[1] : Mitochondrial evolution in the Demospongiae (Porifera): Phylogeny, divergence time, and genome biology. *Molecular Phylogenetics and Evolution* .2021;155: 10.1016/j.ympev.2020.107011 10.1016/j.ympev.2020.107011 33217579

# Reviewer report: Reviewer response for version 1

**DOI:** 10.21956/wellcomeopenres.27280.r131100

**Published:** 2025-09-17

**Authors:** Poppy Hesketh-Best

**Affiliations:** 1 IGTP Institut Germans Trias i Pujol, Barcelona, Spain

**Is the rationale for creating the dataset(s) clearly described?**

 Yes, the rationale is well described and this data note provides a valuable resource to researchers interested in sponge genomics and host-bacterial interactions and symbiosis.

**Are the protocols appropriate and is the work technically sound?**

 I cannot comment on the technical soundless of the sponge genomes assemble, having no experience with those techniques. The bacteria metagenome assembly methods are sound and in following with practices that are commonly utilized today and established.

**Are sufficient details of methods and materials provided to allow replication by others?**

 The authors have provided detailed accounts of the software versions used in their analysis (Table 4). For the host assembly, the workflows are clearly described in a publicly available workflow (BlobToolkit – Nextflow pipeline). However, the same level of detail is not provided for the bacterial metagenome assembly. To strengthen reproducibility, it would be valuable for the authors to additionally provide the working scripts (R, shell, Python, or other languages) used in the analysis for review and critique. 

 It is also unclear whether the 46 MAGs presented in the data note are being made available. If these genomes are not deposited, this should be explicitly stated. At present, no submissions could be identified in the FigShare CSV files, which only contain GTDB classifications and binning completeness/contamination reports, nor is this information clarified in the Data Availability statement. Clarifying the availability of these MAGs is an amendment I would request.

 One important omission is the source and extraction of the host/metagenomic DNA. Was the DNA obtained from a laboratory-maintained sponge or from a wild-caught specimen? This should additionally be clarified.

**Are the datasets clearly presented in a usable and accessible format?**

 Yes the data is currently available. I was able to access the following documents from the data note (2025/09/15)

 - PRJEB72481 accession in ENA

 - PRJEB43743 accession in ENA

 - FigShare repository names: Metagenome of *Diacarnus erythraeanus* (consisting of 2 datafiles)

Are sufficient details of methods and materials provided to allow replication by others?

Partly

Is the rationale for creating the dataset(s) clearly described?

Yes

Are the datasets clearly presented in a useable and accessible format?

Yes

Are the protocols appropriate and is the work technically sound?

Yes

Reviewer Expertise:

microbial ecology, metagenomics, microbial bioinformatics (bacterial, fungal, viral)

I confirm that I have read this submission and believe that I have an appropriate level of expertise to confirm that it is of an acceptable scientific standard, however I have significant reservations, as outlined above.

# Response

**Published:** 2026-03-26

**Authors:** Tree of Life Team Sanger

**Affiliations:** Wellcome Trust Sanger Institute, UK

Thank you for taking the time to review this data note and for your constructive comments. Regarding the two main points raised: the methods for metagenome assembly are described in some detail in the "Metagenome assembly" subsection of the Methods.

 We have now moved this section to appear before Table 4 to improve the flow and make it easier to locate.

 All data have been deposited under the BioProjecthttps://identifiers.org/ena.embl/PRJEB72481, including all raw data, the metagenome assembly, and the assembled MAGs. The metagenome assembly and associated MAGs are held under a child BioProject, accessible at https://www.ebi.ac.uk/ena/browser/view/PRJEB72581. 

 Tree of Life team, Wellcome Sanger Institute
